# Low serum neutralizing anti-SARS-CoV-2 S antibody levels in mildly affected COVID-19 convalescent patients revealed by two different detection methods

**DOI:** 10.1038/s41423-020-00573-9

**Published:** 2020-11-02

**Authors:** Berislav Bošnjak, Saskia Catherina Stein, Stefanie Willenzon, Anne Katrin Cordes, Wolfram Puppe, Günter Bernhardt, Inga Ravens, Christiane Ritter, Christian R. Schultze-Florey, Nina Gödecke, Jörg Martens, Hannah Kleine-Weber, Markus Hoffmann, Anne Cossmann, Mustafa Yilmaz, Isabelle Pink, Marius M. Hoeper, Georg M. N. Behrens, Stefan Pöhlmann, Rainer Blasczyk, Thomas F. Schulz, Reinhold Förster

**Affiliations:** 1 Institute of Immunology, Hannover Medical School, Hannover, Germany; 2 Institute of Virology, Hannover Medical School, Hannover, Germany; 3 Department of Hematology, Hemostasis, Oncology and Stem Cell Transplantation, Hannover Medical School, Hannover, Germany; 4 Institute of Transfusion Medicine and Transplant Engineering, Hannover Medical School, Hannover, Germany; 5 German Primate Center—Leibniz Institute for Primate Research, Göttingen, Germany; 6 Faculty of Biology and Psychology, University Göttingen, Göttingen, Germany; 7 Department of Rheumatology and Immunology, Hannover Medical School, Hannover, Germany; 8 Public Health Department, Hannover, Germany; 9 Department of Pneumology and German Centre of Lung Research (DZL), Hannover Medical School, Hannover, Germany; 10 German Center for Infection Research (DZIF), partner site Hannover-Braunschweig, Hannover, Germany; 11 Cluster of Excellence RESIST (EXC 2155), Hannover Medical School, Hannover, Germany

**Keywords:** COVID-19, SARS-CoV-2, Neutralizing antibody, Serum, ELISA, Viral infection, Antibodies, Infection, Predictive markers

## Abstract

Neutralizing antibodies targeting the receptor-binding domain (RBD) of the SARS-CoV-2 spike (S) block severe acute respiratory syndrome coronavirus 2 (SARS-CoV-2) entry into cells via surface-expressed angiotensin-converting enzyme 2 (ACE2). We used a surrogate virus neutralization test (sVNT) and SARS-CoV-2 S protein-pseudotyped vesicular stomatitis virus (VSV) vector-based neutralization assay (pVNT) to assess the degree to which serum antibodies from coronavirus disease 2019 (COVID-19) convalescent patients interfere with the binding of SARS-CoV-2 S to ACE2. Both tests revealed neutralizing anti-SARS-CoV-2 S antibodies in the sera of ~90% of mildly and 100% of severely affected COVID-19 convalescent patients. Importantly, sVNT and pVNT results correlated strongly with each other and to the levels of anti-SARS-CoV-2 S1 IgG and IgA antibodies. Moreover, levels of neutralizing antibodies correlated with the duration and severity of clinical symptoms but not with patient age. Compared to pVNT, sVNT is less sophisticated and does not require any biosafety labs. Since this assay is also much faster and cheaper, sVNT will not only be important for evaluating the prevalence of neutralizing antibodies in a population but also for identifying promising plasma donors for successful passive antibody therapy.

## Introduction

Within 6 months since its emergence, the novel severe acute respiratory syndrome coronavirus 2 (SARS-CoV-2), the cause of coronavirus disease 2019 (COVID-19), has spread rapidly worldwide. COVID-19 consists of a spectrum of clinical syndromes ranging from asymptomatic cases to mild, flu-like disease to severe illness requiring hospitalization mainly due to pulmonary complications.^1–3^ Although SARS-CoV-2 primarily targets the respiratory system, new data indicate that COVID-19 also affects the vascular system, causing thrombotic microangiopathy and thrombosis in multiple organs, including the lungs.^4–6^ It is not surprising, therefore, that patients with pre-existing cardiovascular diseases, hypertension, and other comorbidities are at particular risk.^7^

SARS-CoV-2 utilizes angiotensin-converting enzyme 2 (ACE2) as a receptor for entry into target cells and employs TMPRSS2, a cellular serine protease, for activation of the viral spike (S) protein.^8,9^ Both ACE2 and TMPRSS2 are abundant in the upper respiratory tract.^10^ An early immune response against SARS-CoV-2 involves interleukin-6 and interferon signature gene expression in alveolar macrophages and infiltrating monocytes.^11^ Although this early immune response is accompanied by severe lymphopenia,^12,13^ increasing data indicate that successful recovery from COVID-19 relies on antibody and T-cell responses.^12,14–17^ Importantly, there appears to be a strong correlation between circulating SARS-CoV-2-specific CD4^+^ and CD8^+^ T cells and IgG antibodies against the nuclear (N) and/or the spike (S) protein of SARS-CoV-2.^16,17^

Current data indicate that anti-SARS-CoV-2 IgM antibodies appear within one week after infection and are present for a month before they gradually decrease.^18,19^ In contrast, anti-SARS-CoV-2 IgG antibodies appear within 10–21 days after infection and appear to remain more-or-less stable for up to 3 months.^18,19^ It is not surprising, therefore, that antibody responses against SARS-CoV-2 have received attention as a method for accurate assessment of infection prevalence.^20,21^ Particularly interesting are antibodies targeting the receptor-binding domain (RBD) of the S protein, as they can block virus entry into cells and thus prevent infection and spread. Furthermore, these neutralizing antibodies may be used for passive antibody therapy,^20,22^ as approved by the United States Food and Drug Administration on March 24th, 2020, as an emergency investigational new treatment for severe or life-threatening COVID-19.^23^

In addition to general safety measures for plasma donation, a crucial parameter in convalescent plasma donor selection for COVID-19 is an adequate neutralizing antibody titer.^24^ However, the field is rapidly evolving, and there is still no consensus about the diagnostic value of divergent ELISA-based antibody tests for SARS-CoV-2 seropositivity.^25^ Moreover, there is uncertainty regarding the durability of anti-SARS-CoV-2 antibody responses, especially as there are indications that antibody responses to other coronaviruses are variable and transient.^26–29^ It is also not clear whether all COVID-19 patients, especially those with mild disease, will produce sufficient amounts of neutralizing antibodies against SARS-CoV-2 to prevent early reinfection.

In the present study, we compared different experimental approaches to qualitatively and quantitatively assess antibody response to SARS-CoV-2 infection primarily in cohorts of convalescent individuals with mild COVID-19 disease. We applied a SARS-CoV-2 S protein-pseudotyped-vesicular stomatitis virus (VSV) vector-based neutralization assay (pVNT)^8^ that has relatively low throughput and relies on infectious viruses, with requirement of a biosafety-2 lab. As reported by others,^30^ we also developed and applied a surrogate virus neutralization test (sVNT) that detects antibodies interfering with the binding of the SARS-CoV-2 S protein RBD to ACE2 in vitro. This assay is based on broadly available ELISA techniques and allows high-throughput analysis. Furthermore, we correlated the functional data obtained by sVNT and pVNT to anti-SARS-CoV-2 S1 IgG and IgA levels measured using a commercial S1 protein ELISA as well as to clinical parameters. We found low titers of neutralizing anti-SARS-CoV-2 S antibodies in 93% of convalescent patients with mild COVID-19. Importantly, only low levels of neutralizing anti-SARS-CoV-2 S titers could be detected in convalescent patients exhibiting clinical symptoms for a short period of time. In contrast, sera from convalescent patients with severe COVID-19 contained significantly higher total and neutralizing antibody titers. Thus, the sVNT allows for high-throughput screening and will be valuable for epidemiological studies as well as for identifying suitable plasma donors for passive immunization.

## Material and methods

### Serum samples

Serum samples were collected from convalescent COVID-19 individuals who volunteered to donate plasma at Hannover Medical School’s (HMS) Institute of Transfusion Medicine and Transplant Engineering. All donors had PCR-diagnosed SARS-CoV-2 infection and showed only mild clinical symptoms. Serum was also collected from inpatients with severe COVID-19 symptoms and from healthy controls without any COVID-19-related symptoms (Tables 1, 2, S1, and S2). The blood donors provided consent prior to blood donation and the inpatients at the time of hospital admission for their samples to be used for research purposes. Written informed consent was obtained from all participants. Studies investigating serum samples from healthy controls and COVID-19 patients were approved by the HMS institutional review board (#9001_BO_K2020, #8973_BO_K2020, and #7901_BO_K2018).

**Table 1:** Main cohort characteristics

Group	*n*	Sex (M/F/ND)	Average age [years (range)]	Average symptom duration [days (range)]	Average sampling post symptom onset [days (range)]
Mild COVID	40	20/19/1	42 (19–68)	10 (0–25)	25 (10–61)
Severe COVID	10	9/1/0	54 (23–67)	36 (19–71)	26 (14–46)
HC	12	3/9/0	39 (25–56)	NA	NA

*HC* healthy controls, *M* male, *F* female, *NA* not applicable, *ND* not disclosed

**Table 2:** Characteristics of the confirmation COVID-19 cohort with mild disease

Group	*n*	Sex (M/F/ND)	Average age [years (range)]	Average symptom duration [days (range)]	Average sampling post symptom onset [days (range)]
Mild COVID	44	29/15/0	47 (23–64)	13 (4–24)	33 (14–64)

*M* male, *F* female, *NA* not applicable, *ND* not disclosed

### ELISA

Serum samples were analyzed in the Clinical Virology Laboratory and Clinic for Rheumatology und Immunology of HMS using the CE-certified versions of Euroimmun SARS-CoV-2 S1 IgG and IgA ELISA (Euroimmun, Lübeck, Germany) according to the manufacturer’s recommendations.

### Pseudotyped virus neutralization assay

A pVNT was performed at HMS’s Institute of Virology and the Primate Center in Göttingen as described earlier.^8^ In brief, pseudotyped VSV particles were produced by calcium-phosphate transfecting HEK293T cells carrying expression plasmids for the respective glycoproteins, either pCAGGS-VSV-G^31^ for expression of VSV-G of the control virus or pCG1-SARS-2 SΔ18^32^ for the SARS-CoV-2 spike protein. Eighteen hours later, the cells were infected with VSV*ΔG-FLuc, a replication-deficient recombinant VSV in which the VSV-G open reading frame has been replaced by combined GFP and firefly luciferase expression cassettes.^33^ This VSV*ΔG-FLuc stock virus was propagated in BHK-21 G43 cells.^34^ After incubating the transduced cells with the viral particles for 2 h at 37 °C, the supernatant was removed, and the cells were washed twice with PBS. The cells were then supplied with medium containing an mAb targeting VSV-G (supernatant from mouse hybridoma CRL-2700; ATCC) to neutralize residual VSV-G, a step that was omitted for the cells transfected with the VSV-G expression construct. Twenty hours later, the pseudotype particle-containing supernatant was separated from the cells by centrifugation and used for neutralization assays.

For the pVNT, Vero76 cells were seeded at 1 × 10^4^ cells per well in 96-well plates. The next day, complement in test sera was inactivated by heating the samples to 56 °C for 30 min. The sera were then serially diluted, mixed 1:2 with the pseudotyped VSV and incubated for 30 min at 37 °C. The medium was removed from the Vero76 cells and replaced in triplicate wells with the serum/pseudotype particle mixture. Twenty hours postinfection, the supernatant was removed from the cells and replaced with 1× luciferase lysis buffer (2× Lysis juice, 102517, PJK). The cells were lysed for 30 min at room temperature. The lysates were transferred to white plates with luciferase substrate (Beetle juice, 102511, PJK), and luciferase activity was measured with a Hidex Sense plate luminometer (Hidex) or a GloMax Discover Microplate Reader (Promega). Data were plotted after background subtraction and normalization to the “no serum” controls. Pseudotyped virus neutralizing titers 50 and 90 (pVNT_50/90_) were defined as the last serum dilution that reduced the transduction efficiency of biological triplicates by at least 50% or 90%, respectively.

### Expression and purification of recombinant soluble ACE2-IgG1 protein

HEK293T cells were grown in DMEM/10% ultralow IgG FBS/PenStrep and transiently transfected with the plasmid pcDNA3-sACE2(WT)-Fc (a gift from Erik Procko; Addgene plasmid #145163^35^) by applying standard calcium-phosphate procedures. Supernatants were collected and separated using a protein A-Sepharose column (ThermoFisher). The bound recombinant protein was eluted with 0.1 M sodium citrate pH 3.5. The buffer was exchanged with PBSd, and integrity as well as purity was confirmed by analyzing 2 µg of protein on a 10% SDS polyacrylamide gel.

### Surrogate virus neutralization assay

The surrogate virus neutralization assay was developed based on the hypothesis that virus neutralizing antibodies should also interfere with the binding of the RBD of SARS-CoV-2 (SARS-CoV-2 S RBD) to soluble, surface-immobilized ACE2, as described by others,^30^ with several modifications. Depending on the amount of neutralizing antibodies present in convalescent sera, the binding of SARS-CoV-2 S RBD to ACE2 should be blocked to various degrees that should correlate with the optical density of this enzyme-linked immune sorbent-based assay. In the assay reported herein, the hACE2 protein (Trenzyme or in-house produced) was coated with different concentrations in 100 mM carbonate-bicarbonate coating buffer (pH 9.6) on F96-Maxisorp Nunc-Immuno plates (Thermo Scientific) at 4 °C overnight. After washing in phosphate-buffered saline (PBS) with 0.05% Tween 20 (PBST), the plates were blocked with 2% bovine serum albumin (BSA, Sigma) and 0.1% Tween 20 in 1× PBS for 1.5 h at 37 °C. Then, His-tag-conjugated SARS-CoV-2 S RBD (Trenzyme) was added to the carrier buffer (1% BSA and 0.05% Tween in 1× PBS) at different concentrations and incubated for 1 h at 37 °C. Unbound SARS-CoV-2 S RBD was removed by four PBST washes before an anti-His peroxidase-labeled monoclonal antibody (mAb; Clone 3D5, prepared in house) in carrier buffer was added for 1 h at 37 °C. After the final wash, the colorimetric signal was developed by adding the chromogenic substrate 3,3′,5,5′-tetramethylbenzidine (Sigma) and stopped by adding an equal volume of stop solution (0.2 M H_2_SO_4_). Finally, absorbance readings at 450 and 570 nm were acquired using the SpectraMax ID3 microplate reader (Molecular Devices). The *K*_D_ values of the SARS-CoV-2 binding affinity to ACE2 were calculated from binding curves based on their global fit using one-site specific binding analysis (GraphPad Prism).

To test for the presence of neutralizing anti-SARS-CoV-2 S serum antibodies, 6 ng of SARS-CoV-2 S RBD was preincubated with test sera at final dilutions between 1:20 and 1:540, as indicated on the graphs, for 1 h at 37 °C before adding them to plates coated with 150 or 300 ng/well ACE2. For each reaction, the percent inhibition was calculated from optical density values after subtraction of background values as: Inhibition (%) = (1 − Sample OD value/Average SARS-CoV-2 S RBD OD value) × 100. Neutralizing sVNT titers were determined as the dilution with binding reduction > mean + 2SD of values from sera of healthy controls.

### Statistical analysis

Linear data were analyzed using an unpaired *t*-test with Welch’s correction (for 2 groups) or Welch’s ANOVA followed by Dunnett’s T3 multiple comparisons test (for 3 groups) and were correlated using the Pearson *r* test. Categorical data were analyzed using the *χ*^2^ test or Fisher’s exact test for unpaired proportions, as indicated beneath each figure. Correlation between linear and categorical data was assessed using ordinary one-way ANOVA followed by the test for linear trend. All statistical analyses were conducted using GraphPad Prism 8.4 (GraphPad Software, USA).

## Results

### Most individuals with mild COVID-19 disease develop anti-SARS-CoV-2 antibodies

Between March 23^rd^ and May 11^th^ 2020, we enrolled as a first step 50 convalescent COVID-19 patients diagnosed with SARS-CoV-2 infection by RT-PCR and 12 healthy control subjects who were not exposed to SARS-CoV-2. The samples from the convalescent COVID-19 cases were split into two groups according to disease severity. Eighty percent of the individuals (*n* = 40) had a mild clinical course, with an average symptom duration of 10 days (range, 0–25 days), and did not require an inpatient hospital stay (Tables 1 and S1). Ten patients had severe COVID-19 and required hospital stays longer than 2 weeks and respiratory support. The patients with severe COVID-19 had an average disease duration of 37 days (range from 19–71 days).

**Fig. 1:**
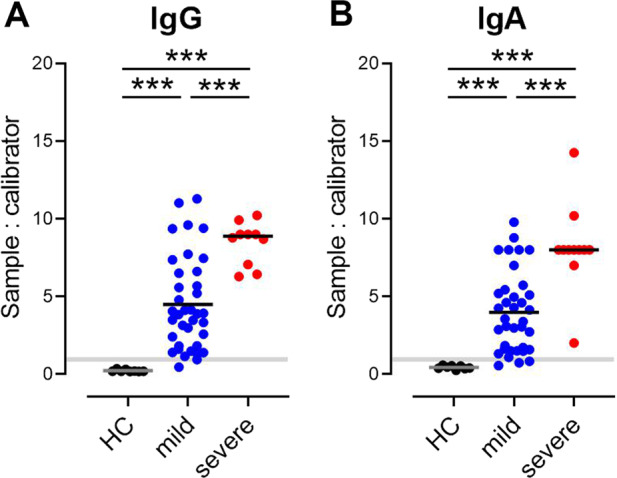
Qualitative analysis of serum total IgG (**A**) and IgA (**B**) antibodies against SARS-CoV-2 S1 in convalescent patients with mild or severe COVID-19 and healthy controls (HC) determined by ELISA. Shaded area cutoff values to determine positive (above), borderline (within), and negative (below gray area) samples. Dots, individuals; bars, mean, ***P < 0.001, Welch’s ANOVA followed by Dunnett’s T3 multiple comparisons test

To estimate overall antibody responses against SARS-CoV-2 in the serum of COVID-19 convalescent individuals, we analyzed the presence of anti-SARS-CoV-2 IgG and IgA antibodies targeting the S1 protein by ELISA. Anti-SARS-CoV-2 S1-specific IgG antibodies were detected in 35/37 (94.6%) of the mildly affected and in 10/10 severely affected COVID-19 patients tested. One individual with mild disease was considered to have borderline serum positivity, and one patient was negative according to the manufacturer’s classification (Fig. 1A). Similarly, anti-SARS-CoV-2 S IgA antibodies were present in 33/37 (89.2%) of the tested sera; two samples were diagnosed as borderline positive and two as negative (Fig. 1B). All sera from tested healthy controls (8/8) were negative for SARS-CoV-2 S-specific IgG and IgA (Fig. 1A, B).

### A surrogate virus neutralization assay for the detection of neutralizing antibodies

**Fig. 2:**
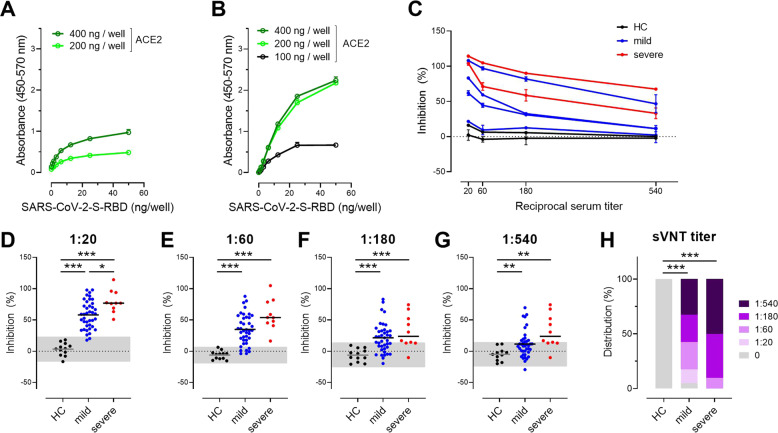
The surrogate virus neutralization test (sVNT) detects neutralizing antibodies interfering with SARS-CoV-2 S RBD binding to human ACE2. Binding of SARS-CoV-2 S RBD to human ACE2 from commercial vendor (**A**) and produced in-house (**B**). Plates were coated with ACE2 as indicated. His-tagged SARS-CoV-2 S RBD was titrated as indicated and detected with an anti-His peroxidase-labeled mAb. Representative assays performed in duplicate are presented as the mean ± SD. **C** Inhibition of the interaction of SARS-CoV-2 S RBD with ACE2 by the addition of sera from convalescent patients with mild (blue lines) or severe (red lines) COVID-19 and healthy controls (HC; black lines). Assay performed in duplicate; mean percentages of neutralization ± SD. **D**–**G** Inhibition of the interaction of SARS-CoV-2 S RBD with ACE2 at the serum dilutions indicated. Individual values (dots) and means (line). Shaded areas represent the mean ± 2SD of values from sera of healthy controls. *P < 0.05; ***P < 0.001, Welch’s ANOVA followed by Dunnett’s T3 multiple comparisons test. **H** Relative distributions of SARS-CoV-2 neutralizing serum titers determined as the dilution retaining binding reduction > mean + 2SD of HC. ***P < 0.001; Fisher’s exact test (HC vs. mild or severe) or the Chi-squared test was used to assess the trend (mild vs. severe)

Based on the notion that serum neutralizing antibodies might also reduce the binding of SARS-CoV-2 to ACE2 in vitro, we developed an sVNT, as reported by others.^30^ We applied the sVNT to determine the levels of neutralizing antibodies in the serum of COVID-19 convalescents and compared them to healthy controls. At the outset, we coated microtiter plates with different amounts of ACE2 obtained from a commercial vendor or produced in-house and titrated soluble His-tagged SARS-CoV-2 S RBD. Binding was revealed by a peroxidase-labeled anti-His mAb and a chromogenic substrate (Fig. 2A, B). The dissociation constant (*K*_D_) in our assay was 5.9 ± 1.9 nM (mean ± SEM) and thus similar to the values reported elsewhere.^36^

Based on the findings, we used 300 ng/well of commercially produced ACE2 or 150 ng/well of in-house produced ACE2 in combination with 6 ng/well SARS-CoV-2 S RBD-His to test sera for their ability to interfere with the binding of these two proteins. Indeed, sera from convalescent patients interfered with S protein binding to ACE2, though with different efficiencies, whereas sera from healthy controls did not (Fig. 2C). Sera from convalescent patients were scored as “positive” at any tested dilution once binding reduction was > the mean + 2SD of values from sera of healthy controls. At serum dilutions of 1:20, this assay identified neutralizing antibodies in 38/40 (95.0%) of mildly affected convalescent patients and in 10/10 of severely affected convalescent patients but in none of the 12 tested healthy control samples (Fig. 2C, D). With increasing serum dilutions, the number of “positive” sera dropped constantly (Fig. 2E–G). The median sVNT titer of the mildly affected convalescent cohort was 1:180, indicating that patients with mild COVID-19 produce relatively low amounts of SASRS-CoV-2 neutralizing antibodies (Fig. 2H). In contrast, severe COVID-19 convalescent patients had a median sVNT titer of ≥1:540 (Fig. 2H). Importantly, data derived from testing several serum samples in assays using two different sources of ACE2 correlated strongly (Fig. S1).

### Neutralizing antibodies determined by a pseudotyped virus neutralization test

**Fig. 3:**
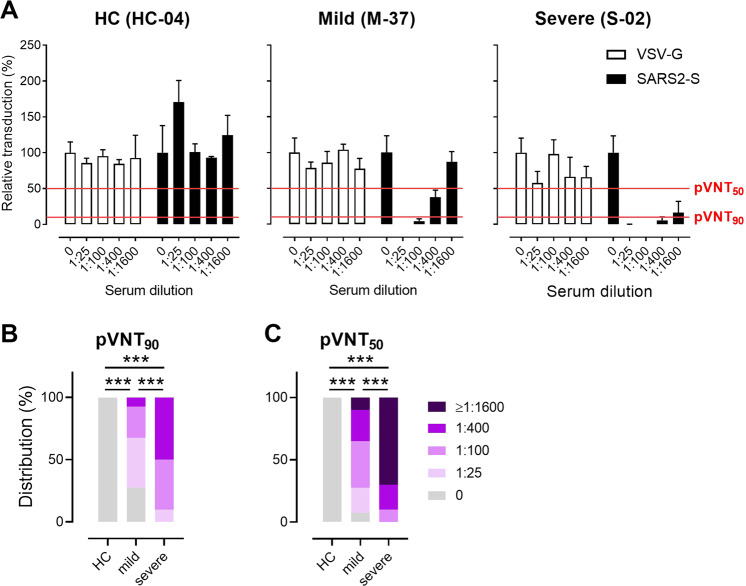
Frequency of neutralizing antibodies against SARS-CoV-2 measured by a pseudotyped virus neutralization test (pVNT) based on SARS-CoV-2 S protein-pseudotyped VSV. **A** Example of pVNT results. Sera from COVID-19 convalescent patients with mild or severe disease—but not from healthy controls (HC)—suppress entry of replication-defective VSV particles carrying the SARS-CoV-2 S protein into host cells (filled bars); neither sera suppressed the entry of control particles carrying the G-protein of VSV entry (open bars). Red lines indicate levels of 50 or 90% suppression of virus entry as indicated. Relative distributions of SARS-CoV-2 neutralizing serum titers that result in (**B**) 90% (pVNT_90_) or (**C**) 50% (pVNT_50_) reduction of luciferase production, as described in (**A**). ***P < 0.001; Fisher’s exact test (HC vs. mild or severe) or the Chi-squared test was used to assess the trend (mild vs. severe)

Next, we determined levels of neutralizing antibodies by an established pVNT that relies on the use of replication-defective VSV particles carrying the SARS-CoV-2 S protein to reflect the entry of SARS-CoV-2 into host cells.^8^ Particles carrying the G-protein of VSV were used as a control. Sera from healthy controls neither suppressed VSV-G nor SARS-CoV-2 S-driven transduction. Similarly, sera from convalescents did not suppress transduction driven by VSV-G but in many cases transduction mediated by SARS-CoV-2 S was inhibited (Fig. 3A). We used this assay to determine the serum dilutions that reduce the transduction of pVSV by 90% (pVNT_90_) and 50% (pVNT_50_) (Fig. 3B, C). In contrast to the 10 tested serum samples from severely affected COVID-19 convalescent patients who all had neutralizing antibodies, such molecules were detected in only 29 (pVNT_90_) and 37 (pVNT_50_) of the 40 tested serum samples from mildly affected convalescents but in none of the sera from healthy controls (Fig. 3B, C). Of note, the median pVNT_50_ in the mildly affected convalescent group was 1:100, and the median pVTN_90_ was 1:25, further supporting the finding that patients with mild COVID-19 only produce low amounts of SARS-CoV-2 neutralizing antibodies. In contrast, the median pVNT_50_ and pVTN_90_ in severely affected COVID-19 convalescents were 1:1600 and 1:400, respectively, further indicating that patients recovering from severe disease produce higher neutralizing anti-SARS-CoV-2 antibody titers than patients with mild disease.

### Positive correlation between total anti-S1 protein and neutralizing antibody levels in sera of convalescent individuals with mild COVID-19

**Fig. 4:**
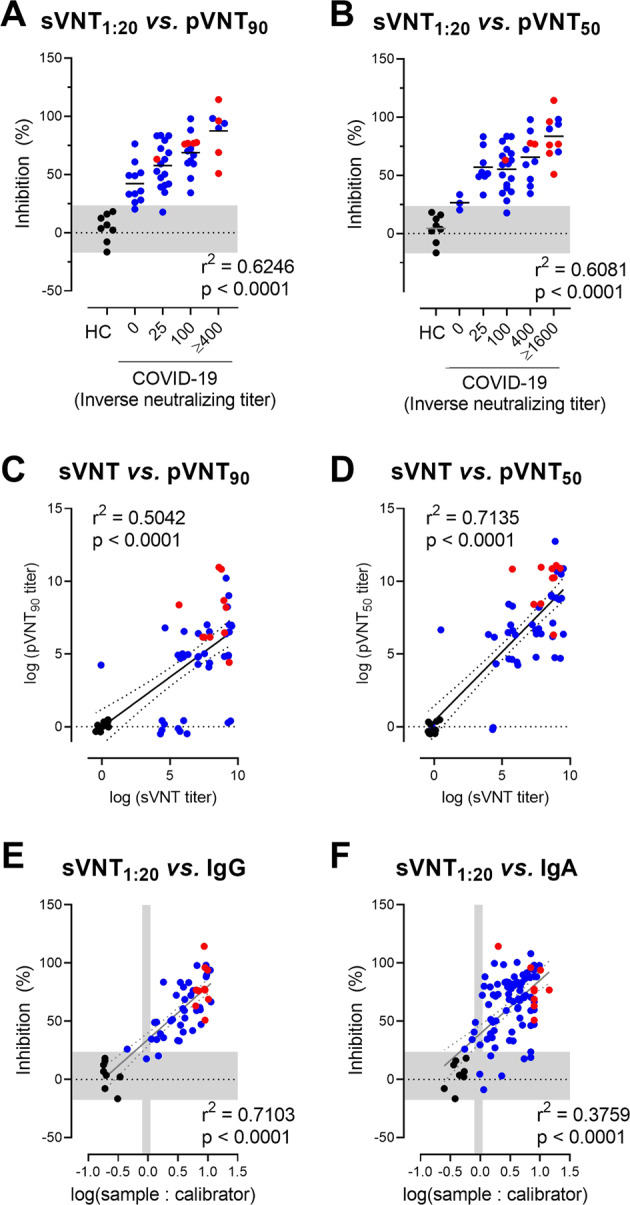
sVNT positively correlates with pVNT and anti-SARS-CoV-2 S1 IgG and IgA antibodies. Correlation between sVNT_1:20_ and antibody titers resulting in 90% (**A**) or 50% (**B**) reduction of luciferase production in pVNT_90_ and pVNT_50_. The horizontal shaded area indicates the mean ± 2SD range of inhibition of sera from HC. Correlation between log-transformed sVNT titers (determined as in Fig. 2H) and log-transformed pVNT_90_ (**C**) and pVNT_50_ (**D**). To alleviate overplotting, the titer values were jittered by the addition of random values in the interval [−0.5, 0.5]. Correlation between sVNT_1:20_ and log-transformed SARS-CoV-2 S1-specific IgG (**E**) and IgA (**F**) levels measured by ELISA. The vertical shaded areas indicate the respective cutoff values recommended by the manufacturer to determine positive (right to), borderline (within) and negative (left to) shaded areas. The horizontal shaded area indicates the mean ± 2SD range of inhibition of sera from healthy controls. **A**–**F** Dots, samples of HC (black), mildly (blue), or severely (red) affected COVID-19 convalescent cases. **C**–**F** Linear correlation (solid line) and 95% confidence intervals (dotted lines). Correlation, one-way ANOVA followed by a test for the trend (**A**, **B**) or Pearson *r* (**C**–**F**)

We then analyzed the correlation between total levels of anti-S1 IgG and IgA and the amount of neutralizing antibodies in our cohort of mild COVID-19 convalescent patients and healthy controls. As expected, an initial comparison showed a strong positive correlation between the levels of S protein-specific IgA and IgG antibody in sera (Fig. S2). More importantly, there was a robust positive correlation between the percent inhibition of SARS-CoV-2 S RBD binding to ACE2 at a 1:20 serum dilution (sVNT_1:20_) and pVNT_90_ as well as pVNT_50_ inhibitory titers (Fig. 4A, B). Furthermore, there was a strong positive correlation between sVNT and pVNT_50_ as well as pVNT_90_ inhibitory titers (Fig. 4C, D).

Similarly, a strong positive correlation between sVNT_1:20_ and levels of SARS-CoV-2 S1-specific IgG and antibody levels in convalescents and healthy controls was observed, though levels of anti-S1 IgA showed a weaker correlation (Fig. 4E, F). These data demonstrate that sVNT reliably detects neutralizing serum antibodies against SARS-CoV-2. Furthermore, a strong correlation with *r*^2^ values between 0.64 and 0.75 was revealed when comparing SARS-CoV-2 S1-specific IgG and IgA antibody levels with pVTN_90_ and pVTN_50_ (Fig. S3).

### Total and neutralizing anti-SARS-CoV-2 S antibody levels in sera correlate positively with symptom duration but not with the timing of sampling or patient age

**Fig. 5:**
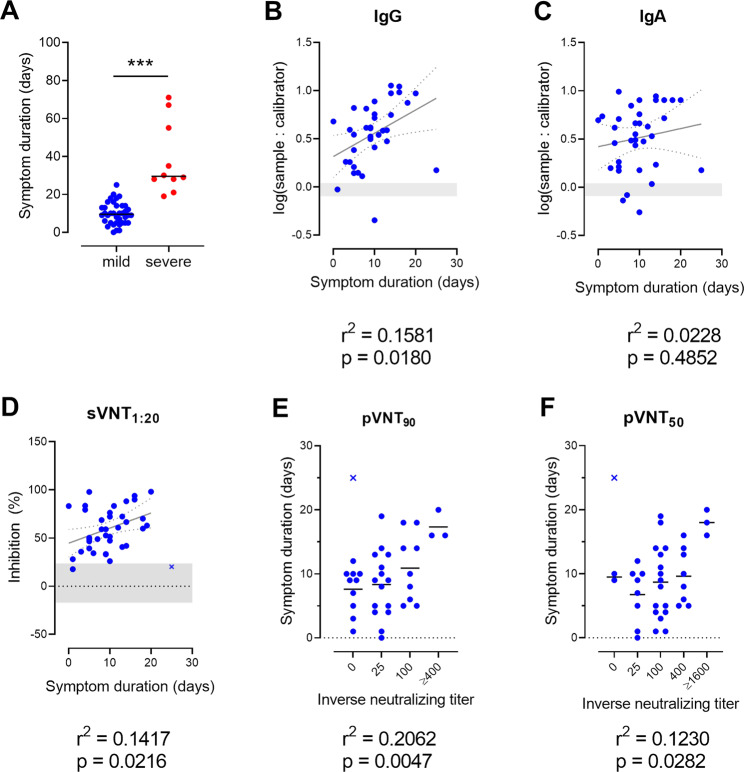
The duration of symptoms correlates with total and neutralizing SARS-CoV-2 S-specific antibody levels. **A** Symptom duration of mild and severely affected COVID-19 patients. Dots, individuals; bars, mean, ***P < 0.001, Welch’s *t* test. Weak positive correlation between duration of symptoms and levels of log-transformed SARS-CoV-2 S1-specific IgG (**B**) and IgA (**C**) antibodies, sVNT_1:20_ (**D**), pVNT_90_ (**E**), or pVNT_50_ (**F**) neutralizing antibody titers. Dots, convalescent individuals with mild COVID-19, outliers are marked with *x*, horizontal lines, means. **B**, **C** Shaded areas indicate vendor-defined cutoff values to determine positive (above), borderline (within), and negative (below gray area) samples. **D** The shaded area indicates the mean ± 2SD range of inhibition of sera from HC. Correlation, Pearson *r* (**B**–**D**) or one-way ANOVA followed by a test for the trend (**E**, **F**). An outlier was defined as a value with absolute residual value > 2SD of all residual values (**D**) or as a value > mean ± 2SD of values with the same titer

We next examined whether the level of the protective humoral response to SARS-CoV-2 in COVID-19 patients correlated with disease duration, as defined by the number of days patients showed symptoms (mild cases) or until they were discharged from the hospital (severe cases). Not surprisingly, severely affected patients had a 3.5 times longer disease duration, averaging 36 days, than the 10 days of the mildly affected patients (Fig. 5A). As severely ill patients produced higher neutralizing and total antibody titers, these data indicate that disease duration might directly influence antibody titers. This hypothesis is further supported by a positive correlation between the duration of symptoms and total anti-SARS-CoV-2 IgG, but not IgA, antibodies in convalescent patients with mild disease (Fig. 5A, B). Although weaker, there was also a positive correlation between symptom duration and levels of neutralizing antibodies, as determined by sVNT_1:20_, pVTN_90_, and pVTN_50_ (Fig. 5D, E). These data are in agreement with data reported by Robbiani et al.^37^ and with a recent publication indicating that asymptomatic SARS-CoV-2 infection induces lower antibody levels than symptomatic infection.^38^ Altogether, our results and publicly available data suggest that a certain threshold of disease severity and/or duration might be required for successful mounting of neutralizing anti-SARS-CoV-2 humoral responses.

Conversely, in our cohort of convalescent COVID-19 cases, we did not observe any correlation between levels of total and neutralizing anti-SARS-CoV-2 S antibodies with the timing of sampling after the occurrence of first symptoms (Fig. S4). Similarly, the levels of neutralizing anti-SARS-CoV-2 S IgG and IgA antibodies did not correlate with the age of patients who had recovered from mild disease, even though convalescent patients with severe disease were on average older than those with mild COVID-19 (Fig. S5). Of note, our cohort of convalescent COVID-19 patients with mild disease was selected based on the ability to donate blood and therefore included only three elderly patients (aged 60 or over). Thus, there is not sufficient power to detect levels of total and neutralizing anti-SARS-CoV-2 S IgG and IgA antibodies in this age group. Similarly, most of the severely affected patients investigated in this study were males, but the relatively small cohort size did not allow analysis of sex associations using our data.

### sVNT allows rapid screening of sera of blood donors for the presence of neutralizing antibodies

**Fig. 6:**
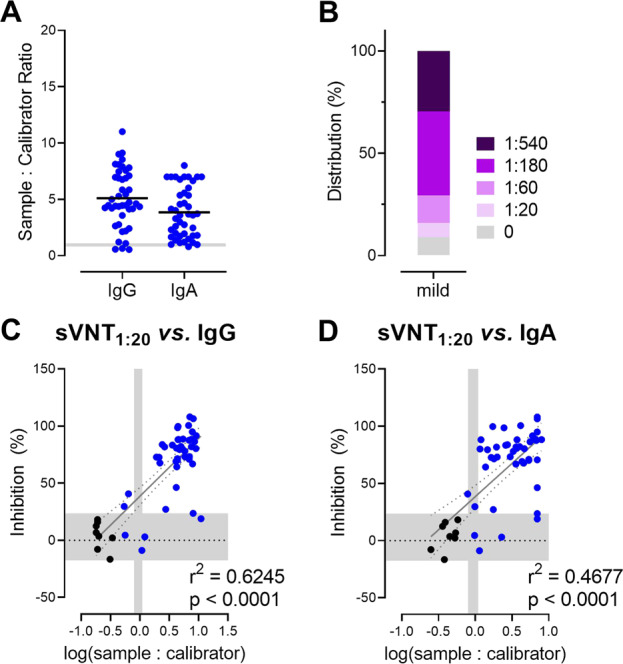
sVNT correlates positively with total levels of anti-S IgG and IgA antibodies against SARS-CoV-2 S1 in a validation cohort of convalescent patients with mild COVID-19 (*n* = 44). **A** Serum anti-SARS-CoV-2 S1 antibodies determined by ELISA. Shaded areas, cutoff values to determine positive (above), borderline (within), and negative (below gray area) samples. Dots, individuals; bars, mean. **B** Relative distribution of SARS-CoV-2 neutralizing serum titers determined as the dilution retaining binding reduction > mean + 2SD of HC. Correlation between sVNT_1:20_ and log-transformed SARS-CoV-2 S1-specific IgG (**C**) and IgA (**D**) levels measured by ELISA. Vertical shaded areas, vendor-defined cutoff values to determine positive (right to), borderline (within), and negative (left to shaded area) samples. Horizontal shaded area, the mean ± 2SD inhibition of sera from HC. Correlation, Pearson *r*

To validate our findings, we recruited a second cohort of 44 convalescent patients with mild COVID-19 and analyzed their sera by ELISA and sVNT (Tables 2 and S2). In this group of patients, ELISA detected S1 protein-specific IgA and IgG antibodies in 38 of 44 analyzed serum samples (borderline counted as negative; Fig. 6A). As described above for the first group of mildly affected convalescent patients, levels of S protein-specific IgA and IgG antibodies showed a strong correlation (Fig. S6). Similarly, applying the sVNT confirmed that mildly affected COVID-19 convalescent patients possess relatively low titers of neutralizing anti-S RBD antibodies (median 1:180). This assay revealed neutralizing antibodies in the sera of 40 of 44 (90.9%) individuals with mild COVID-19 (Fig. 6B). Along the same line, a strong positive correlation between the sVNT_1:20_ and total levels of SARS-CoV-2 S-specific IgG and IgA antibody levels was also identified (Fig. 6C, D). Moreover, in this group of samples, we observed a weak positive correlation between symptom duration and sVNT_1:20_ or log-transformed SARS-CoV-2 S-specific IgG but not IgA levels (Fig. S7A–C). As expected, we did not detect any correlation between specific IgG, IgA, or sVNT_1:20_ and patient age or date of sampling (Fig. S7D–F and data not shown). As a final validation, we correlated the sVNT_1:20_ and total levels of SARS-CoV-2 S-specific IgG and IgA antibody levels, pooling the data from the initial and validation cohorts. As depicted in Fig. S8, the pooled set of data revealed a strong positive correlation between sVNT_1:20_ and total levels of SARS-CoV-2 S-specific IgG and IgA antibody levels as well as a weak positive correlation between symptom duration and sVNT1:20 and log-transformed SARS-CoV-2 S-specific IgG but not IgA levels.

Altogether, the results from the validation cohort confirmed our initial results and further emphasize the usefulness of the sVNT for rapid screening of a larger number of samples for the presence of neutralizing anti-SARS-CoV-2 S RBD antibodies.

## Discussion

A detailed understanding of immune responses following SARS-CoV-2 infection will enable better treatment and diagnostic procedures, as well as the development of successful vaccines that will help to control the global COVID-19 pandemic. In this regard, it is important to gain a better understanding of the presence of neutralizing anti-SARS-CoV-2 serum antibodies in the population, as they potentially prevent (re)infection and might be a treatment option.^39,40^ As reported by others,^30^ we established an sVNT that is based on ELISA technology and thus can be adapted to allow for high-throughput analysis of samples. We validated this assay by comparing data from the sVNT with those derived from a classical pVNT and found a strong correlation between the results obtained with these two tests, which is in line with the results of Tan et al.^30^ Our experience confirms that sVNT is technically less complicated, cheaper, and much faster than pVNT, making it more suitable for the rapid screening of a large number of samples. Of note, Tan et al. also showed that sVNT, apart from a small degree of cross-neutralization with anti-SARS-CoV antibodies, is specific for SARS-CoV-2.^30^ Standardization of this test might, therefore, be important for the selection of convalescent plasma donors for the treatment of COVID-19 patients. Furthermore, as this assay does not rely on antispecies antibodies, it can also be applied to detect neutralizing antibodies in any animal species used for preclinical testing of SARS-CoV-2 vaccines. sVNT might also be adapted for the detection of immunoglobulin classes that most efficiently neutralize SARS-CoV-2 S or used for rapid screening of neutralizing capacities of monoclonal SARS-CoV-2 S RBD-specific antibodies, accelerating the development of drugs based on recombinant neutralizing antibodies. On the other hand, a disadvantage of sVNT, as compared to pVNT, is its intrinsic inability to detect neutralizing antibodies other than those binding to the SARS-CoV-2 S RBD. Nevertheless, these non-RBD-targeting antibodies appear to have only a minor role in SARS-CoV-2 neutralization,^37,41^ which is supported by the robust correlation between sVNT and pVNT reported in the present study and by others.^30^

Combining pVNT and sVNT, we found that ~90% of recovered patients with mild COVID-19 possessed neutralizing serum antibodies. These findings are in line with an early report suggesting that recovered COVID-19 patients have neutralizing SARS-CoV-2 S RBD antibodies in serum after discharge from the hospital;^16^ other preliminary data indicate that neutralizing SARS-CoV-2 S RBD antibodies are undetectable in one-third of convalescent COVID-19 patients.^37,42^ Additional studies are therefore required to provide more detailed insight into the levels of neutralizing SARS-CoV-2 S antibodies in convalescent COVID-19 individuals from different countries. Those studies should also exclude false-positive PCR or COVID-19 misdiagnosis as the possible reason for the lack of antibodies in certain suspected COVID-19 patients. This would be particularly important, as convalescent COVID-19 individuals without neutralizing antibodies might still be susceptible to reinfection and would not be able to provide plasma for the prevention and treatment of COVID-19.

Our data also corroborate other studies indicating that levels of neutralizing antibodies in convalescent COVID-19 individuals are generally low.^37,42^ Interestingly, Robbiani et al. reported recently that individual neutralizing antibodies against SARS-CoV-2 S RBD have a half-maximal inhibitory concentration against authentic SARS-CoV-2 ranging from 3 to 709 ng/ml.^37^ Together, these data suggest that a considerable proportion of COVID-19 patients with mild disease can produce intermediate- to high-affinity IgG antibodies. Since such antibodies are not present in a certain proportion (5–30%) of COVID-19 cases, these findings indicate that effective virus clearance does not rely exclusively on the humoral response but also includes cellular immune responses.^12,14–17^ The present investigation was performed primarily on young and middle-aged convalescent COVID-19 patients with mild disease fulfilling all criteria for blood donation. It is therefore not surprising that in the present study, patient age did not affect neutralizing anti-SARS-CoV-2 S IgG titers, as has been reported by others.^37,42^ Our data indicate that serum levels of total and neutralizing anti-SARS-CoV-2 S antibodies correlate positively with the duration of symptoms, which is also in line with the observations made by others.^42^ This hypothesis is further supported by findings that significantly higher titers of neutralizing antibodies were present in sera of severely affected than in sera of mildly affected patients. However, as another study did not find a correlation between the duration of symptoms and neutralizing anti-SARS-CoV-2 S IgG titers,^37^ future studies are needed to evaluate how age or disease duration affects neutralizing antibody titers.

The passive transfer of donor plasma seems to provide clinical benefit for severe but not critically ill patients.^24,40^ Encouraged by the promising results from initial studies with small patient numbers, more than 50 larger randomized clinical trials are currently evaluating the effectiveness of plasma transfusion from convalescent to ill COVID-19 patients.^24^ The results of these studies will provide an answer as to whether the success of passive immunization by plasma transfusion correlates to total or neutralizing antibody levels in donor sera. Moreover, the results will also be important for the development and potential clinical application of recombinant neutralizing anti-SARS-CoV-2 antibodies, such as the recently described 47D11.^43^

In conclusion, this study reports a high-throughput sVNT for SARS-CoV-2. The results obtained with this assay correlate highly with data obtained by classical but laborious and time-consuming pVNT. Both assays revealed the presence of neutralizing anti-SARS-CoV-2 S antibodies, albeit at low titers, in the sera of many but not all convalescent COVID-19 patients with mild disease. Although these findings have implications for the selection of convalescent donors for passive immunization by plasma therapy, additional studies are required to understand why neutralizing anti-SARS-CoV-2 S antibodies do not develop in all patients and how long neutralizing antibodies are present in patients who have recovered from COVID-19.

## Supplementary information

Supplementary data
