# Supplementary material for: Low serum neutralizing anti-SARS-CoV-2 S antibody levels in mildly affected COVID-19 convalescent patients revealed by two different detection methods
Source: Cell Mol Immunol. 2020 Nov 2;18(4):936–44. doi: 10.1038/s41423-020-00573-9 (PMC7604543; doi:10.1038/s41423-020-00573-9)
Supplement: Supplementary file 1 — Supplementary data [file 41423_2020_573_MOESM1_ESM.docx]

Low serum neutralizing anti-SARS-CoV-2-S antibody levels in mildly affected COVID-19 convalescent patients revealed by two different detection methods

Berislav Bošnjak^1,*^, Saskia Catherina Stein^2,^, Stefanie Willenzon^1^, Anne Katrin Cordes^2^, Wolfram Puppe^2^, Günter Bernhardt^1^, Inga Ravens^1^, Christiane Ritter^1^, Christian R. Schultze-Florey^1,3^, Nina Gödecke^4^, Jörg Martens^4^, Hannah Kleine-Weber^5,6^, Markus Hoffmann^5,6^, Anne Cossmann^7^, Mustafa Yilmaz^8^, Isabelle Pink^9^, Marius M. Hoeper^9^, Georg M.N. Behrens^7,10^, Stefan Pöhlmann^5,6^, Rainer Blasczyk^4^, Thomas F. Schulz^2,10,11^, Reinhold Förster^1,11,*^

^1^ Institute of Immunology, Hannover Medical School, Hannover, Germany

^2^ Institute of Virology, Hannover Medical School, Hannover, Germany

^3^ Department of Hematology, Hemostasis, Oncology and Stem Cell Transplantation, Hannover Medical School, Germany

^4^ Institute of Transfusion Medicine and Transplant Engineering, Hannover Medical School, Hannover, Germany

^5^ German Primate Center – Leibniz Institute for Primate Research, Göttingen, Germany

^6^ Faculty of Biology and Psychology, University Göttingen, Göttingen Germany

^7^ Department of Rheumatology and Immunology, Hannover Medical School, Hannover, Germany

^8^ Public Health Department, Hannover, Germany

^9^ Department of Pneumology and German Centre of Lung Research (DZL), Hannover Medical School, Germany

^10^ German Center for Infection Research (DZIF), partner site Hannover-Braunschweig, Hannover, Germany

^11^ Cluster of Excellence RESIST (EXC 2155), Hannover Medical School, Hannover, Germany

**Running title:** Antibodies in COVID-19 convalescent patients

**Supplementary Information**

Supplementary figures S1 – S8

Supplementary tables S1 and S2

**Figure S1.** Strong positive correlation of percentage inhibition determined by two different sources of ACE2. Samples from mild (blue, n=10) and severe (red, n=4) COVID-19 convalescent patients and HC (black dots, n=4) were analyzed. Correlation, Pearson r.


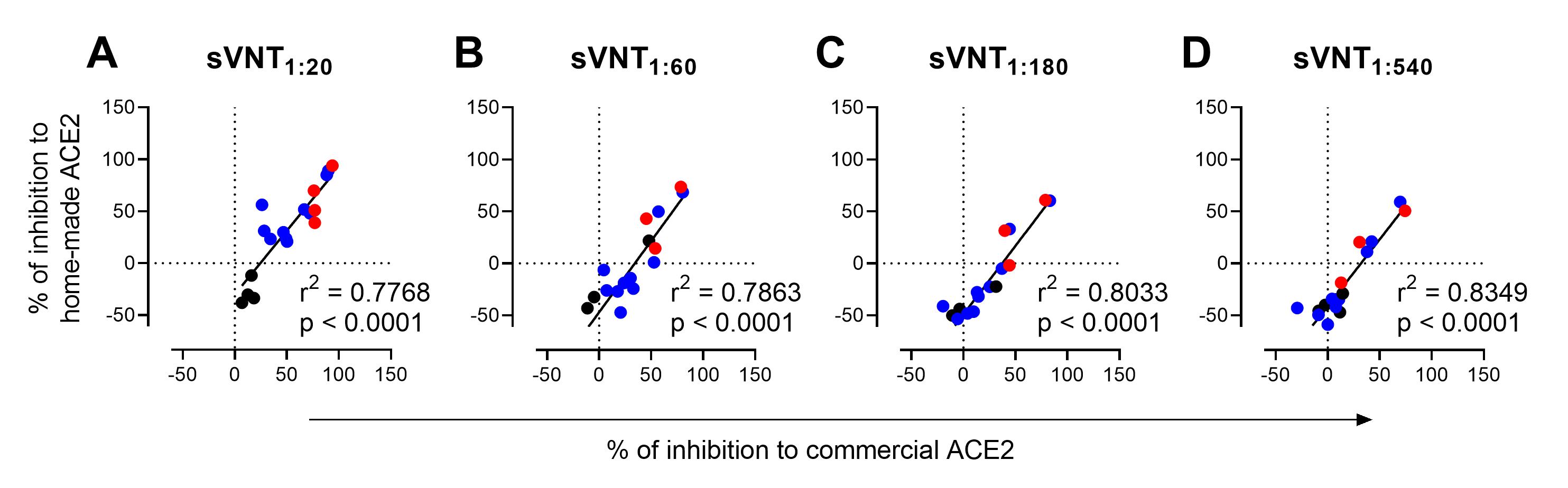


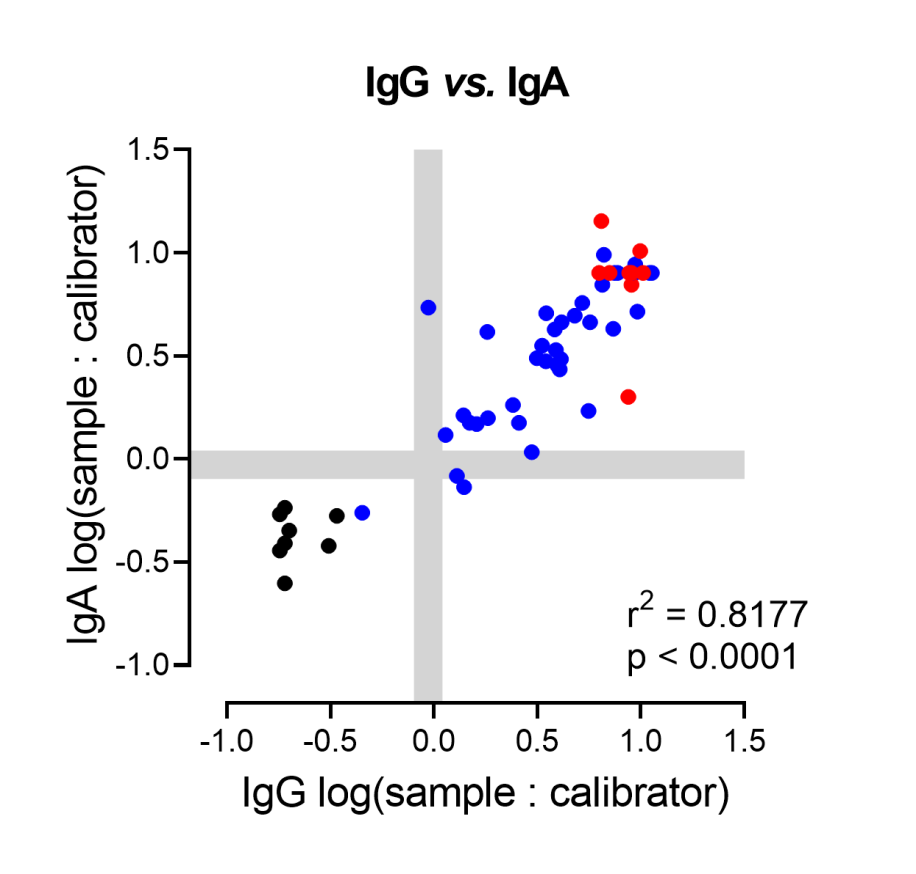


**Figure S2.** Total IgG and IgA antibody levels against SARS-CoV-2 strongly correlate. Samples from mild (blue) and severe (red) COVID-19 convalescent patients and HC (black dots). Shaded areas, vendor-defined cut-off values to determine positive, borderline and negative samples as described for figure 1. Correlation, Pearson r.


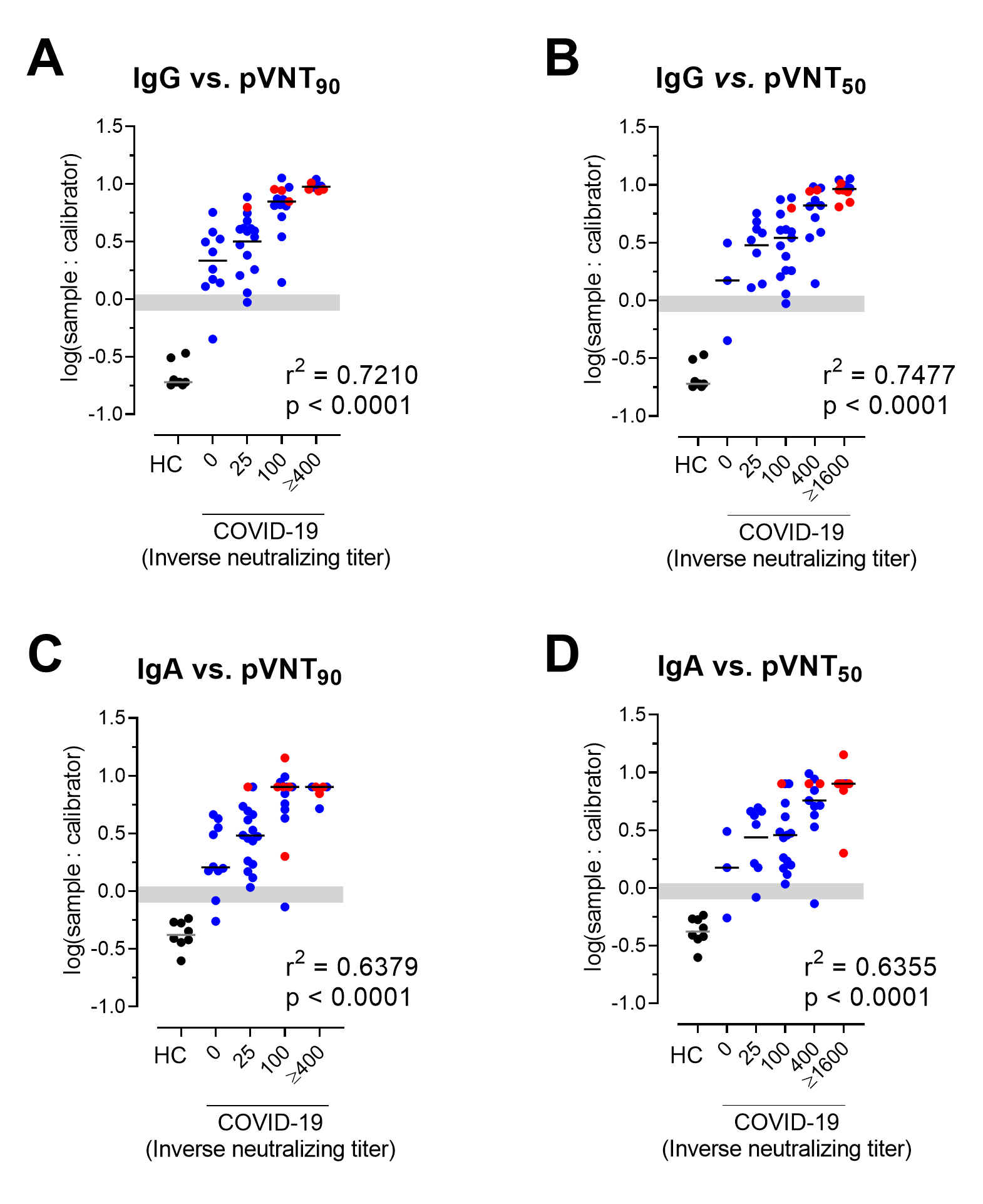


**Figure S3.** Positive correlation between pVNT and anti SARS-CoV-2-S1 IgG and IgA antibodies

(A-D) Anti SARS-CoV-2-S1-specific IgG and IGA levels determined by ELISA and pVNT90 or pVNT50 neutralizing antibody titers as indicated. Dots, samples of HC (black), mildly (blue) or severely (red) affected COVID-19 convalescent patients. Shaded areas indicate cut-off values to determine positive (above), borderline (within) and negative (below gray area) samples. Correlation, one-way ANOVA followed by test for trend.


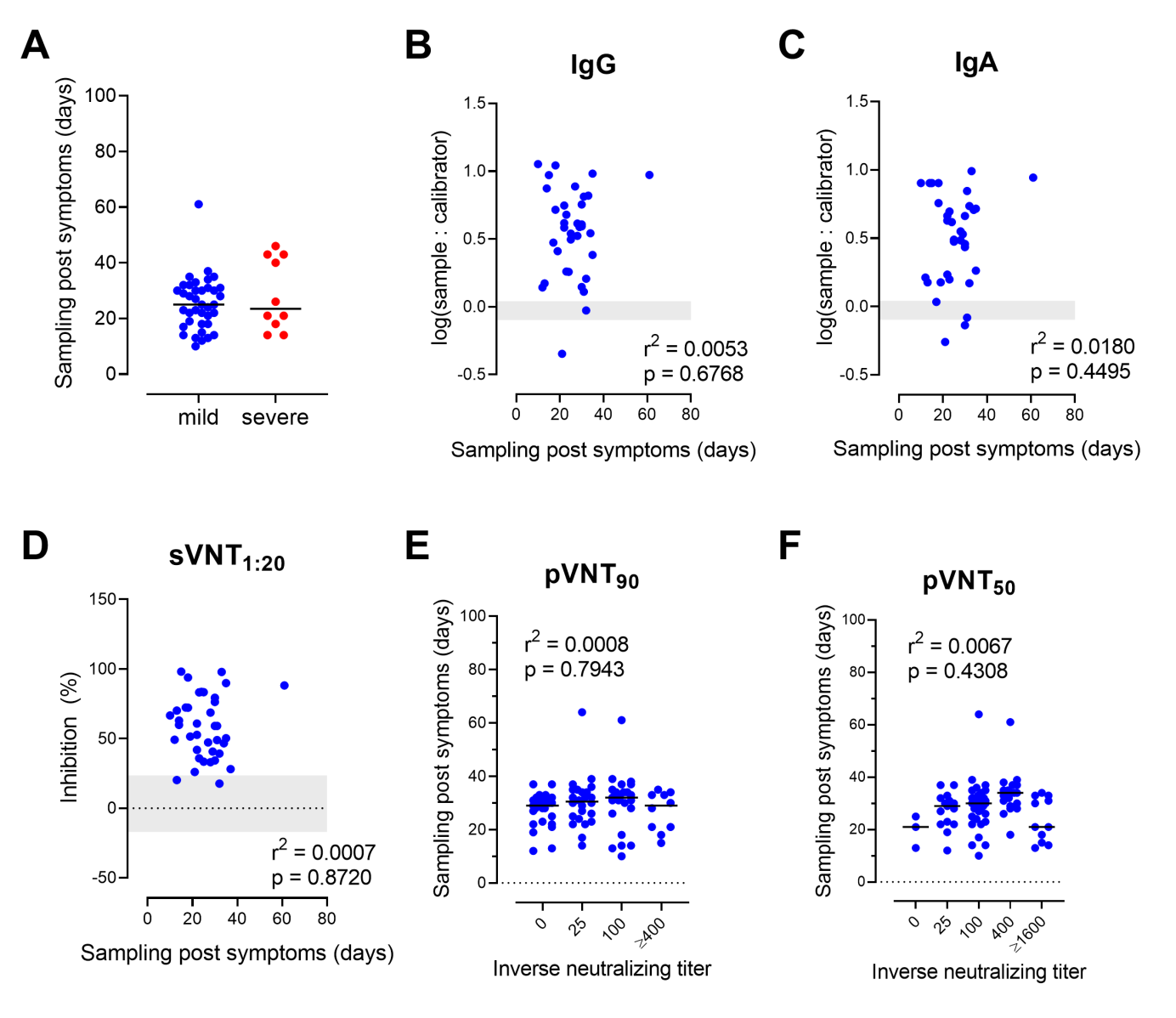


**Figure S4.** No correlation between time of sampling and total and neutralizing SARS-CoV-2-specific antibody levels**.**

(A) No difference between date of sampling between convalescent patients with mild and severe COVID-19. Dots, individuals; bars, mean, * P < 0.05, Welch’s t test.

(B-F) No correlation between time of sampling (post occurrence of symptoms) and log-transformed SARS-CoV-2-S1-specific IgG (B) and IgA (C) levels, sVNT1:20 (D), pVNT90 (E) or pVNT50 (F) in convalescent patients with mild COVID-19. Dots, individuals, (E, F) horizontal lines, medians. (B,C) Shaded areas indicate vendor-defined cut-off values to determine positive (above), borderline (within) and negative (below gray area) samples. (D) The shaded area indicates mean ± 2 SD range of inhibition of sera from HC; Correlation, Pearson r (B-D) or one-way ANOVA followed by test for trend (E,F).


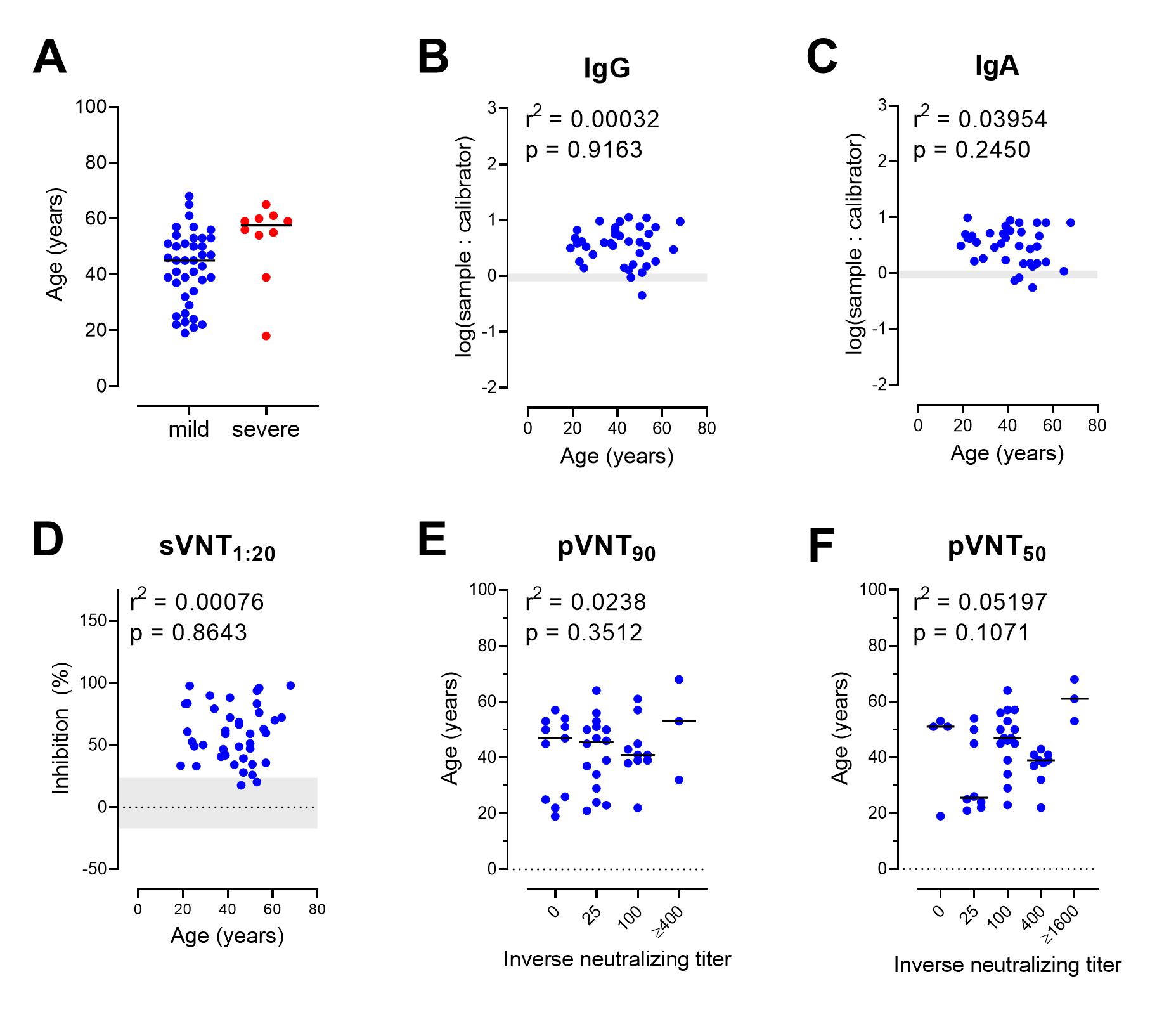


**Figure S5.** No correlation between patient age and total and neutralizing SARS-CoV-2-S-specific antibody levels.

(A) Convalescent patients with severe COVID-19 were significantly older than patients with mild disease. Dots, individuals; bars, mean, * P < 0.05, Welch’s t test.

(B-F) No correlation between age and log-transformed SARS-CoV-2-S-specific IgG (B) and IgA (C) levels, sVNT1:20 (D), pVNT90 (E) or pVNT50 (F). Dots, individuals recovered from mild COVID-19

(E, F) horizontal lines, medians. (B,C) Shaded areas indicate vendor-defined cut-off values to determine positive (above), borderline (within) and negative (below gray area) samples. (D) The shaded area indicates mean ± 2 SD range of inhibition of sera from HC; Correlation, Pearson r (B-D) or one-way ANOVA followed by test for trend (E,F).


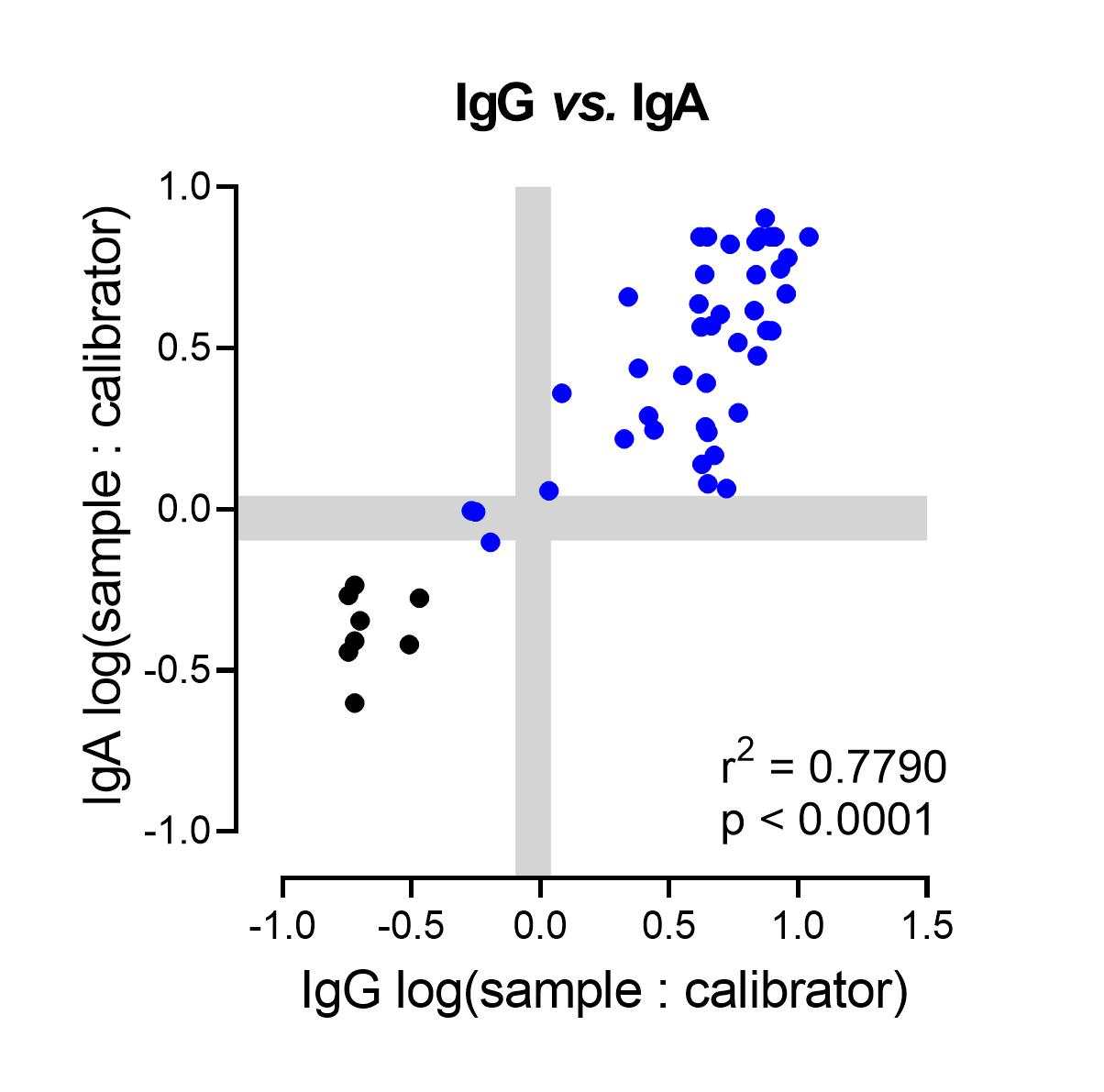


**Figure S6.** Total IgG and IgA antibody levels against SARS-CoV-2-S1 strongly correlate in the validation group. Samples from mild (blue) COVID-19 convalescent patients and the initial HC (black dots). Shaded areas: vendor-defined cut-off values to determine positive, borderline and negative samples as described for figure 1. Correlation, Pearson r.


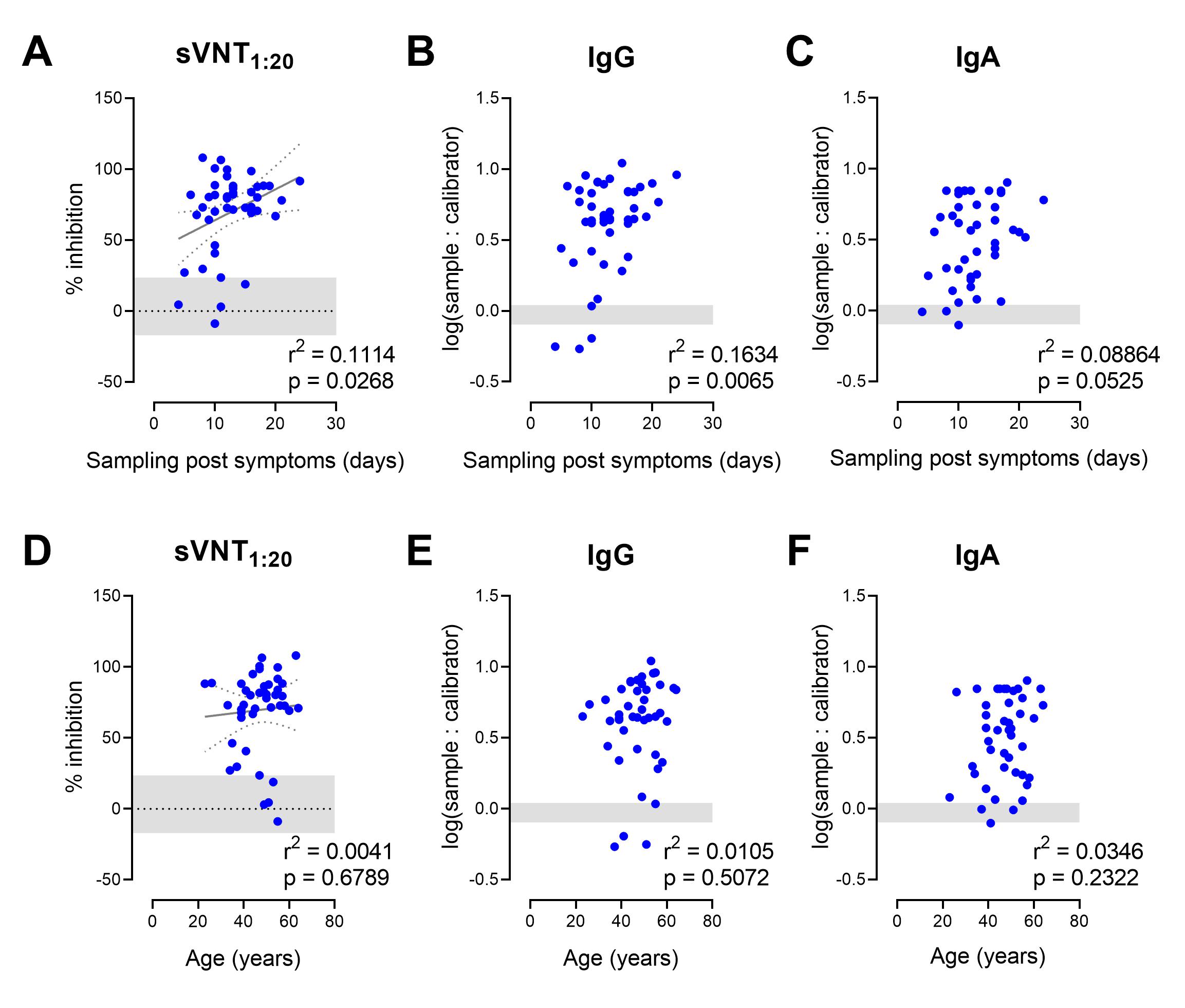


**Figure S7.** Duration of symptoms but not patient age positively correlates to total and neutralizing SARS-CoV-2-S-specific antibody levels in the validation group of convalescent patients with mild COVID-19

(A-C) Weak positive correlation between duration of symptoms and sVNT_1:20_ (A) and log-transformed SARS-CoV-2-S1-specific IgG (B) but not IgA (C) levels.

(D-F) No correlation between age of patients recovered from mild COVID-19 and sVNT_1:20_ (G) or log-transformed anti SARS-CoV-2-S1-specific IgG (H) and IgA (I) antibody levels.

Dots, individuals; horizontal lines, means. Shaded areas indicate mean ± 2 SD of inhibition of sera from HC (A,D,G) or the respective cut-off values from ELISA as described for Fig 5 (B,C,E,F,H,I). Correlation, Pearson r (A-C,G-I); Comparison Welch’s t test (D-F).


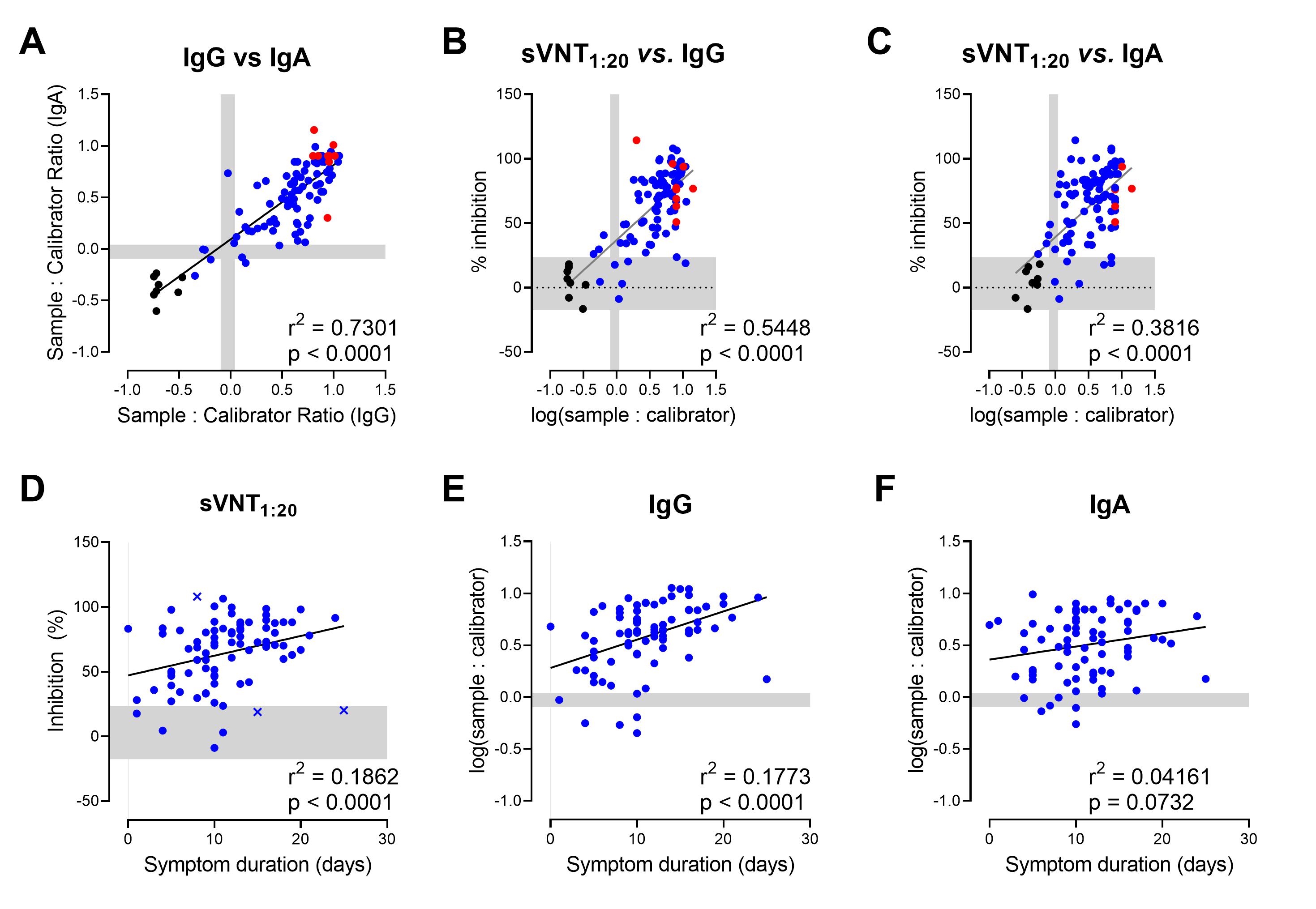


**Figure S8. C**orrelation of sVNT with anti SARS-CoV-2-S1 IgG and IgA antibodies, and the duration of symptoms with total and neutralizing SARS-CoV-2-S-specific antibody levels in the pooled (primary and validation) patient cohort.

(A-C) Positive correlation between serum IgG and IgA antibody levels against SARS-CoV-2-S1 (A) as well as between sVNT_1:20_ and log-transformed SARS-CoV-2-S1-specific IgG (B) but not IgA (C) levels measured by ELISA. Dots, serum of HC (black), mildly (blue) or severely (red) affected COVID-19 convalescent patients Shaded areas: (A) vendor-defined cut-off values to determine positive, borderline and negative samples as described for figure 1 or (B,C) the respective cut-off values recommended by the manufacturer (vertical areas) or mean ± 2 S.D. range of inhibition of sera from healthy controls (horizontal areas).

(D-F) Weak positive correlation between duration of symptoms and sVNT_1:20_ (D) and levels of log-transformed SARS-CoV-2-S1-specific IgG (E) but not IgA (F) antibodies. Dots, convalescent individuals with mild COVID-19, x marks the outliers, defined as a value with absolute residual value > 2SD of all residual values (D). The shaded areas indicate: (D) mean ± 2 SD range of inhibition of sera from HC or (E,F) vendor-defined cut-off values to determine positive (above), borderline (within) and negative (below gray area) samples.

(A-F) Correlation, Pearson r.

**Table S1.** Demographic and clinical characteristics of individuals with mild and severe COVID-19 and healthy controls (HC). Abbreviations: PEMC - pre-existing medical conditions; m: male; f: female; NA: not available; *: in days; §: days after onset of symptoms.

| ID | Gender | Age | PEMC | COVID-19 | Symptoms | ECMO or intubation | Symptom duration^*^ | Sampling^§^ |
| --- | --- | --- | --- | --- | --- | --- | --- | --- |
| M-01 | F | 20-25 | no | mild | NA | no | 5 | 12 |
| M-02 | M | 45-50 | no | mild | Fatigue, cough, ageusia, body aches, headache | no | 14 | 10 |
| M-03 | M | 50-55 | no | mild | NA | no | NA | NA |
| M-04 | NA | 60-65 | no | mild | Limb pain | no | 18 | 13 |
| M-05 | M | 65-70 | yes | mild | Fever, chills, cough, body aches, sore throat, | no | 20 | 15 |
| M-06 | f | 65-70 | no | mild | Fever, Sore throat, diarrhea (once) | no | 13 | 17 |
| M-07 | M | 20-25 | no | mild | No symptoms | no | 0 | 23 |
| M-08 | F | 55-60 | no | mild | Cough, chills | no | 3 | 23 |
| M-09 | M | 55-60 | yes | mild | Cough, fever, headache, body aches | no | 18 | 14 |
| M-10 | F | 55-60 | no | mild | Chills, fever, diarrhea | no | 19 | 14 |
| M-11 | F | 20-25 | no | mild | Cough, headache, body aches, swollen lymph nodes (4 days) | no | 9 | 22 |
| M-12 | M | 40-45 | no | mild | fever, cough | no | 10 | 18 |
| M-13 | M | 50-55 | no | mild | Fatigue, cough, headache, anosmia, body aches | no | 16 | 18 |
| M-14 | F | 50-55 | no | mild | Cough, anosmia, rhinitis, ageusia | no | 10 | 21 |
| M-15 | F | 35-40 | no | mild | Mild cough | no | NA | NA |
| M-16 | F | 50-55 | no | mild | Body ache (2 days), loss of smell | no | 10 | 27 |
| M-17 | M | 25-30 | no | mild | Rhinitis | no | 5 | 35 |
| M-18 | M | 50-55 | yes | mild | Cough, fever | no | 25 | 13 |
| M-19 | F | 50-55 | no | mild | Headache, shortness of breath, pressure on the bronchi | no | 10 | 30 |
| M-20 | M | 15-20 | no | mild | Cough, fever | no | 9 | 25 |
| M-21 | M | 50-55 | no | mild | Fever, headache, lung pain | no | 10 | 19 |
| M-22 | M | 20-25 | no | mild | Fatigue, cough | no | 12 | 22 |
| M-23 | F | 40-45 | no | mild | Light symptoms | no | 7 | 31 |
| M-24 | F | 25-30 | no | mild | Mild fever, cough, fatigue, chills | no | 9 | 28 |
| M-25 | M | 35-40 | no | mild | Cough, sore throat, fever | no | 8 | 31 |
| M-26 | M | 50-55 | no | mild | Diarrhea, cough, rhinitis, fever | no | 9 | 30 |
| M-27 | F | 35-40 | no | mild | Fever, headache, fatigue | no | 14 | 22 |
| M-28 | M | 45-50 | no | mild | Cough | no | 1 | 32 |
| M-29 | M | 35-40 | no | mild | Fever, body aches, anosmia, ageusia | no | 5 | 34 |
| M-30 | F | 45-50 | no | mild | Headache, fatigue | no | 1 | 37 |
| M-31 | F | 40-45 | yes | mild | Cough, headache, dyspnea | no | 6 | 30 |
| M-32 | F | 40-45 | yes | mild | Cough, fatigue | no | 8 | 28 |
| M-33 | F | 45-50 | no | mild | Fever, light cough, bedridden for 2 days | no | 5 | 32 |
| M-34 | M | 35-40 | no | mild | Fever, chills, fatigue, cough, running nose | no | 13 | 29 |
| M-35 | M | 30-35 | no | mild | Sore throat, elevated temperature, cough, fatigue | no | 4 | 30 |
| M-36 | M | 20-25 | no | mild | Fever, sore throat | no | 5 | 33 |
| M-37 | F | 20-25 | no | mild | Fever, body aches, rhinitis, sore throat | no | 4 | 24 |
| M-38 | F | 50-55 | no | mild | Fever, dyspnea, headache | no | 11 | 25 |
| M-39 | M | 40-45 | NA | mild | Body aches, ageusia, chills | no | 14 | 61 |
| M-40 | F | 30-35 | no | mild | Fever, diarrhea, body aches, headache, pneumonia, cough | no | 16 | 35 |
| S-01 | F | 50-55 | no | severe | Fever, body aches, dyspnea, dizziness | intubation | 28 | 21 |
| S-02 | M | 55-60 | NA | severe | Dyspnea, fever | intubation | 35 | 21 |
| S-03 | M | 60-65 | yes | severe | Fever, dyspnea, pneumonia, cough | intubation | 30 | 26 |
| S-04 | M | 50-55 | NA | severe | Fever, dyspnea, body aches, ageusia, chills | intubation | 29 | 40 |
| S-05 | M | 60-65 | no | severe | Fever, diarrhea, dry cough | ECMO | 71 | 43 |
| S-06 | M | 50-60 | yes | severe | Dyspnea, fever, dry cough | intubation | 67 | 46 |
| S-07 | M | 35-40 | no | severe | Dyspnea, fever | intubation | 55 | 43 |
| S-08 | M | 65-70 | yes | severe | Dyspnea, fever | ECMO | 28 | 14 |
| S-09 | M | 55-60 | yes | severe | Dyspnea, fever | intubation | 19 | 14 |
| S-10 | M | 15-20 | no | severe | Loss of smell, fever, nausea, emesis, dysnpea | no | 21 | 18 |
| S-01 | F | 50-55 | no | severe | Fever, body aches, dyspnea, dizziness | intubation | 28 | 21 |
| HC-01 | F | 35-40 | NA | NA | NA | NA | NA | NA |
| HC-02 | F | 30-35 | NA | NA | NA | NA | NA | NA |
| HC-03 | F | 35-40 | NA | NA | NA | NA | NA | NA |
| HC-04 | F | 20-25 | NA | NA | NA | NA | NA | NA |
| HC-05 | F | 50-55 | NA | NA | NA | NA | NA | NA |
| HC-06 | F | 45-50 | NA | NA | NA | NA | NA | NA |
| HC-07 | M | 20-25 | NA | NA | NA | NA | NA | NA |
| HC-08 | M | 45-50 | NA | NA | NA | NA | NA | NA |
| HC-09 | F | 55-60 | NA | NA | NA | NA | NA | NA |
| HC-10 | F | 30-35 | NA | NA | NA | NA | NA | NA |
| HC-11 | F | 50-55 | NA | NA | NA | NA | NA | NA |
| HC-12 | M | 30-35 | NA | NA | NA | NA | NA | NA |

**Table S2.** Clinical details of confirmation mild COVID-19 cases. Abbreviations: M –male; F- female; PEMC - pre-existing medical conditions; NA: not available; *: in days; §: days after onset of symptoms.

| ID | Gender | Age | PEMC | COVID-19 | Symptoms | ECMO or intubation | Symptom duration^*^ | Sampling^§^ |
| --- | --- | --- | --- | --- | --- | --- | --- | --- |
| M-41 | M | 60-65 | no | mild | fever, diarrhea, anosmia, ageusia | no | 8 | 34 |
| M-42 | F | 40-45 | yes | mild | headache, body aches, anosmia, ageusia, fever (light), rhinitis | no | 13 | 27 |
| M-43 | M | 35-40 | NA | mild | dyspnea, fever, headache, sore throat | no | 10 | 33 |
| M-44 | F | 55-60 | yes | mild | cough, rhinitis, slightly elevated temperature | no | 12 | 28 |
| M-45 | M | 45-50 | no | mild | cough, fatigue (5 days), pressure on the lungs | no | 11 | 33 |
| M-46 | M | 35-40 | no | mild | fever, headache, diarrhea, chill, cough, anosmia, ageusia | no | 8 | 30 |
| M-47 | M | 55-60 | no | mild | cough (light), rhinitis | no | 16 | 31 |
| M-48 | M | 25-30 | no | mild | rhinitis, anosmia, ageusia | no | 10 | 31 |
| M-49 | M | 45-50 | no | mild | fever, joint pain, bitter taste, pneumonia,headaches | no | 10 | 26 |
| M-50 | M | 50-55 | no | mild | cough (light), tiredness, loss of taste | no | 9 | 33 |
| M-51 | F | 45-50 | no | mild | increased temperature, snuff (light), loss of taste | no | 6 | 39 |
| M-52 | M | 40-45 | yes | mild | cough (light), aching limbs, headaches | no | 16 | 34 |
| M-53 | M | 40-45 | yes | mild | fever, aching limbs, loss of smell and taste, cough | no | 20 | 28 |
| M-54 | F | 40-45 | no | mild | headaches, cough, weld outbreaks, fever | no | 17 | 36 |
| M-55 | F | 45-50 | yes | mild | aching limbs, respiratory problems, loss of smell, fever, cough, fatigue | no | 21 | 34 |
| M-56 | M | 45-50 | no | mild | fever,fatigue,loss of smell, passed out (one time), diarrhoea | no | 11 | 37 |
| M-57 | M | 45-50 | yes | mild | fever (light), cough (light), aching limbs | no | 13 | 37 |
| M-58 | M | 45-50 | no | mild | fever, sore throat, headaches, fatigue, loss of smell and taste | no | 17 | 30 |
| M-59 | F | 55-60 | yes | mild | cough,nausea, aching limbs, restricted sense of smell | no | 12 | 37 |
| M-60 | M | 60-65 | no | mild | fever | no | 16 | 38 |
| M-61 | M | 45-50 | no | mild | fever, aching limbs, loss of smell and taste, cough | no | 13 | 35 |
| M-62 | F | 40-45 | no | mild | fever, aching limbs, cough | no | 12 | 39 |
| M-63 | M | 55-60 | no | mild | cough drier, fever, snuff, shivering, headaches, loss of smell and taste, severe fatigue, light bronchitis | no | 24 | 37 |
| M-64 | M | 50-55 | no | mild | loss of smell and taste, diarrhoea, dry cough, loss of appetite | no | 15 | 34 |
| M-65 | M | 35-40 | no | mild | fever (light), fatigue | no | 9 | 64 |
| M-66 | F | 30-35 | n.a. | mild | keine Symptome eingetragen | no | 8 | 32 |
| M-67 | M | 35-40 | no | mild | fever, cough, sore throat, shivering, apathy,fatigue | no | 10 | 29 |
| M-68 | M | 50-55 | no | mild | Cough, fever (1 day), headache, body aches | no | 17 | 26 |
| M-69 | M | 50-55 | no | mild | fatigue, chills, body aches | no | 12 | 32 |
| M-70 | F | 55-60 | no | mild | chills, fever (light), headache, fatigue, body aches | no | 15 | 29 |
| M-71 | M | 45-50 | yes | mild | fever, cough, headache, dyspnea | no | 10 | 30 |
| M-72 | F | 55-60 | yes | mild | Headache, sore throat, anosmia, ageusia, tinnitus | no | 12 | 34 |
| M-73 | F | 35-40 | no | mild | conjunctivitis, cough, fever,anosmia, ageusia, diarrhea | no | 19 | 31 |
| M-74 | F | 60-65 | yes | mild | cough, sore throat, headache, fatigue, vertigo, blood pressure fluctuation | no | 16 | 35 |
| M-75 | F | 20-25 | no | mild | fever, fatigue, body aches, cold symptoms | no | 13 | 31 |
| M-76 | F | 50-55 | no | mild | headache, body aches, ageusia | no | 13 | 33 |
| M-77 | M | 45-50 | yes | mild | fever, fatigue, anosmia, ageusia | no | 16 | 32 |
| M-78 | M | 55-60 | yes | mild | cough, headache, body aches, fever (light) | no | 18 | 14 |
| M-79 | F | 40-45 | no | mild | fever, cough, ageusia, rhinitis | no | 10 | 28 |
| M-80 | M | 35-40 | yes | mild | no symptoms | no | 7 | 31 |
| M-81 | M | 45-50 | yes | mild | cold symptoms | no | 11 | 22 |
| M-82 | M | 50-55 | no | mild | cough, ageusia | no | 10 | 32 |
| M-83 | M | 30-35 | no | mild | slightly elevated temperature, sore throat, cough, fatigue | no | 5 | 32 |
| M-84 | M | 50-55 | no | mild | aegusia (very briefly), nausea for a few days in the morning | no | 4 | 30 |
